# Luteolin inhibits cell proliferation and induces cell apoptosis via down-regulation of mitochondrial membrane potential in esophageal carcinoma cells EC1 and KYSE450

**DOI:** 10.18632/oncotarget.15832

**Published:** 2017-03-02

**Authors:** Ping Chen, Jing-Yang Zhang, Bei-Bei Sha, Yan-Er Ma, Tao Hu, Yang-Cheng Ma, Hao Sun, Jian-Xiang Shi, Zi-Ming Dong, Pei Li

**Affiliations:** ^1^ Cancer Chemoprevention Collaborative Innovation Center in Henan Province, Zhengzhou University, Zhengzhou, Henan, 450001, China; ^2^ Department of Pathophysiology, College of Basic Medical Sciences, Zhengzhou University, Zhengzhou, Henan, 450001, China; ^3^ College of Public Health, Henan Key Laboratory for Tumor Epidemiology, Affiliated Tumor Hospital, Zhengzhou University, Zhengzhou, Henan, 450001, China

**Keywords:** luteolin, esophageal squamous carcinoma, cell proliferation, mitochondrial membrane potential, chemotherapy

## Abstract

In current study, we investigated the anti-tumor effect of luteolin in human ESCC cell lines *in vitro* and *in vivo* and tried to explore the potential mechanisms. Results from flow cytometry showed that luteolin could induce apoptosis and caspase-3 activation and induce cell cycle arrest at G2/M phase in a dose- and time-dependent manner in EC1 and KYSE450 cells. JC-1 test results showed that membrane potential of mitochondria after luteolin treatment was down-regulated and this was an indicator for intrinsic apoptosis. Western Blot results showed the expression of cell cycle regulatory protein p21 and p53 increased and three apoptosis related proteins that participate in mitochondrial apoptotic pathway, namely, Bim, CYT-c and cPARP, also increased in luteolin treated cells compared with control groups. We further confirmed that luteolin could significantly inhibit the growth of ESCC tumors in xenograft mouse models and no evidence of systemic toxicity was observed. Our results suggest that luteolin can induce cell apoptosis and cell cycle arrest in G2/M phase through mitochondrial pathway in EC1 and KYSE450 cell lines and proper utilization of luteolin might be a practical approach in ESCC chemotherapy.

## INTRODUCTION

Esophageal squamous cell carcinoma (ESCC), the predominant histological subtype of esophageal cancer, and it is characterized by high mortality and striking geographic variation throughout the world with the highest incidence rate in east Asia [1, 2]. ESCC is characterized with high metastatic rate, high mortality rate and poor prognosis. Generally, the 5-year survival rate for esophageal cancer that localized only in esophagus is 40% while the rate for esophageal cancer with distant metastasis is only 4%, and the overall 5-year survival rate for esophageal cancer is less than 20% [3]. Most ESCC patients present with advanced metastatic disease because people tend to seek for medical assistance after symptoms develop [4]. Currently, surgical resection is curative only at an early stage in ESCC and chemoradiotherapy is recommended for majority of patients with advanced esophageal cancer [5–7]. Traditional chemotherapy drugs, such as cisplatin, carboplatin, have certain effects on ESCC, at the same time the regimen have a high incidence of side effects, such as mucositis and leukocytopenia, as well as treatment-related death (16%) [8]. It is important to find chemotherapeutic agents with higher specificity, efficacy and fewer side effects for ESCC.

Luteolin is a kind of natural plant flavonoid, which can be derived from a variety of vegetables, fruits, and medicinal herbs in glycosidic form [9]. Like other flavonoids, luteolin possesses many pharmacological properties including antioxidant, anti-tumor, anti-inflammatory, cardio protective, and immune regulation [10, 11]. Among these properties, anti-tumor activity has attracted more attention. Researchers have shown that luteolin has anti-tumor activities in several types of cancer cell lines, including apoptosis induction, cell cycle arrest, metastasis and angiogenesis inhibition in several different types of human cancer cell lines [12–14]. The anti-proliferation effect is associated with flavonoid induced cell cycle arrest, either at the G1/S or G2/M checkpoint [11, 15–19]. Many researchers have also tried to explored the underlying mechanisms [14, 20–23]. Zhang *et al* reported that luteolin can induce G2/M arrest in both KYSE510 ESCC and OE33 EAC cell lines [17, 18]. Wang *et al* reported that luteolin can induce G0/G1 cell cycle arrest in Eca109 human ESCC cell line *in vitro* [19]. And these mechanisms might contribute to its anti-tumor effects. However, the anti-tumor activities in human esophageal cancers needs to be validated *in vitro* and *in vivo*.

Thus, we designed current study to focus on the cell cycle arrest and cell apoptotic effect of luteolin on esophageal cancer cells *in vitro* and try to explore the underlying mechanisms. Moreover, we investigated the anticancer potential of luteolin *in vivo* in ESCC xenograft mouse models.

## RESULTS

### Luteolin inhibited proliferation and growth of EC1, EC9706, KYSE30 and KYSE450 cells *in vitro*

**Figure 1:**
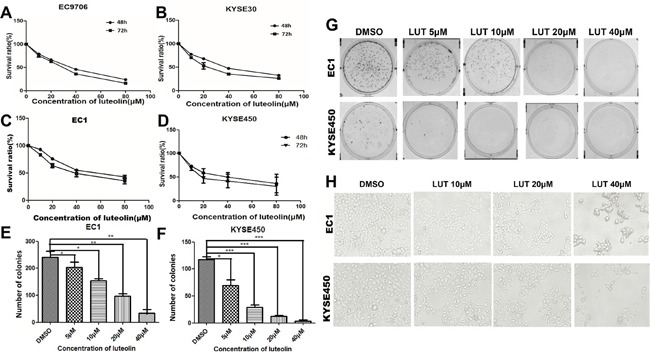
Luteolin inhibited cell proliferation and growth in ESCC cells **(A-D)** Different ESCC cells were exposed to different concentrations of luteolin (0, 10, 20, 40, 80 μM) for 48 h and 72h and then cell viability was measured the by CCK-8 assay. **(E)** and **(F)** Colony count of EC1 and KYSE 450 cells after being treated with luteolin for 8 d. Plate colony formation assay showed that luteolin could reduce the number of EC1 and KYSE450 cell colonies in a dose-dependent manner. **(G)** Representative images of cell colonies after being treated with different concentrations of luteolin for 8 d. **(H)** Representative morphological changes under the invert microscope after EC1 and KYSE450 cells being treated with different concentrations of luteolin (×200). The experiments were repeated three times. (*p < 0.05, **p < 0.01).

As shown in Figure 1, the CCK-8 assay showed that luteolin could suppress the growth of these cells in a dose-dependent manner, compared to the control group (Figure 1A, 1B, 1C and 1D, both *p* < 0.05). Considering the degree of differentiation and cell origins, we chose EC1 and KYSE450 cell lines in further experiments. The half maximal inhibitory concentration (IC_50_) fell in 20 and 60 μM range in these cell lines. We chose 20 and 40 μM as experimental concentrations in further experiments to avoid severe cytotoxic side effect. Plate colony formation assay showed that different concentrations of luteolin could reduce the number of EC1 and KYSE450 cell colonies compared with control groups. Colony-forming efficacies of EC1 and KYSE450 cells were compromised with the increase of concentration of luteolin. Both colony numbers (*p* < 0.05) and in colony sizes decreased (Figure 1E, 1F and 1G). Moreover, morphological changes were also observed under the invert microscope in EC1 and KYSE450 cells after cells being treated with different concentrations of luteolin for 72 h. Most of the cells had lost regular shape, cell junctions disappeared and cell adhesion decreased, cells could easily detach from the substrate after the plates were slightly shaken (Figure 1H). With the concentration of luteolin increased, floating dead cells and cell debris increased. No evidence of microbe or pathogen contamination was observed.

### Luteolin induced cell cycle arrest with up-regulation of the cell cycle inhibitory proteins p21 and p53 in ESCC cells

**Figure 2:**
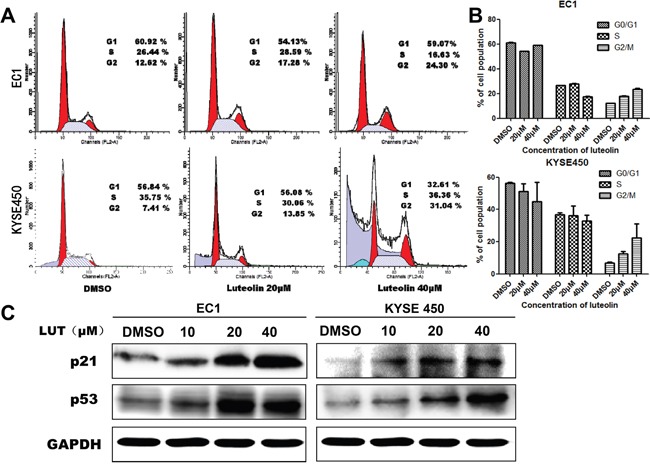
Luteolin induced the cell cycle arrest in EC1 and KYSE450 cells **(A)** DNA contents were analyzed by flow cytometry after EC1 and KYSE450 cells being treated with different concentrations of luteolin (0, 20, 40 μM) for 24 h; **(B)** The percentage of cells in the G0/G1, S, and G2/M phases of the cell cycle were calculated. Results are presented as mean ± SD from three independent experiments. **(C)** Expression of p21 and p53 after EC1 and KYSE450 cells being treated with different concentrations of luteolin for 72 h. GAPDH was used as reference.

Several studies have demonstrated that luteolin could induce cell cycle arrest in different types of cancer cell lines, which can further lead to programmed cell death. The effect of luteolin on cell apoptosis was investigated by flow cytometry. The results show that luteolin induced cell growth inhibition EC1 and KYSE450 cells. Cell population increased in the G2/M phase but decreased in the S phase in a dose-dependent manner both in EC1 and KYSE450 cells when compared with control group (*p <* 0.05, Figure 2A and 2B). Moreover, Western Blotting results show that with luteolin concentration increased, the expression of p21 and p53 proteins also increased (Figure 2C). Our data indicated that luteolin inhibited cell proliferation by blocking cells in G2/M phase and this process is associated with up-regulation of the cell cycle inhibitory proteins p21 and p53.

### Luteolin induced apoptosis via activating caspase-3 in EC1 and KYSE450 cells

**Figure 3:**
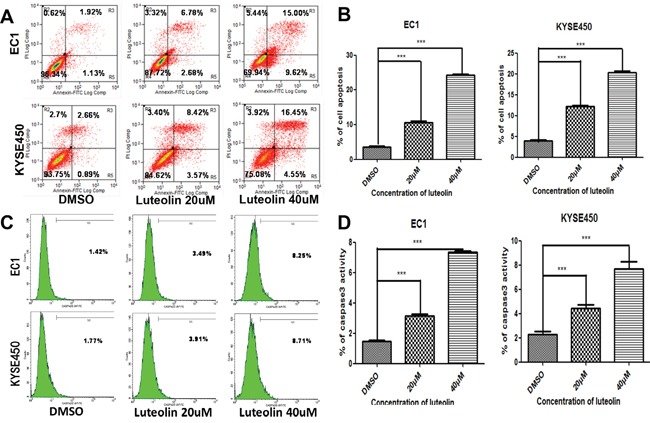
The effect of luteolin on cell apoptosis and caspase-3 activation were investigated by flow cytometry **(A)** Apoptotic rate was analyzed by flow cytometry after EC1 and KYSE450 cells being treated with different concentrations of luteolin (0, 20, 40 μM) for 72 h. **(B)** The percentage of cell apoptosis after EC1 and KYSE450 cells being treated with different concentrations of luteolin were calculated. **(C)** and **(D)** Caspase-3 activation were investigated by flow cytometry. All results are expressed as mean ± SD from three independent experiments. (***p < 0.001).

The effect of luteolin on cell apoptosis was further investigated by flow cytometry. The apoptotic rates at 72 h after different treatments are shown in Figure 3A. The total apoptotic rates (including early and late stages apoptotic rates) for EC1 and KYSE450 cells increased when compared with control groups (both *p* < 0.05, Figure 3B). As shown in Figure 3C and 3D, higher activity of caspase3 in EC1 and KYSE450 cells was associated with higher luteolin concentrations (both *p* < 0.05). These results indicated that luteolin could induce cell apoptosis via activating caspase-3.

### Luteolin could decrease mitochondrial membrane potential via up-regulation of Bim, CYT-C and cPARP protein

**Figure 4:**
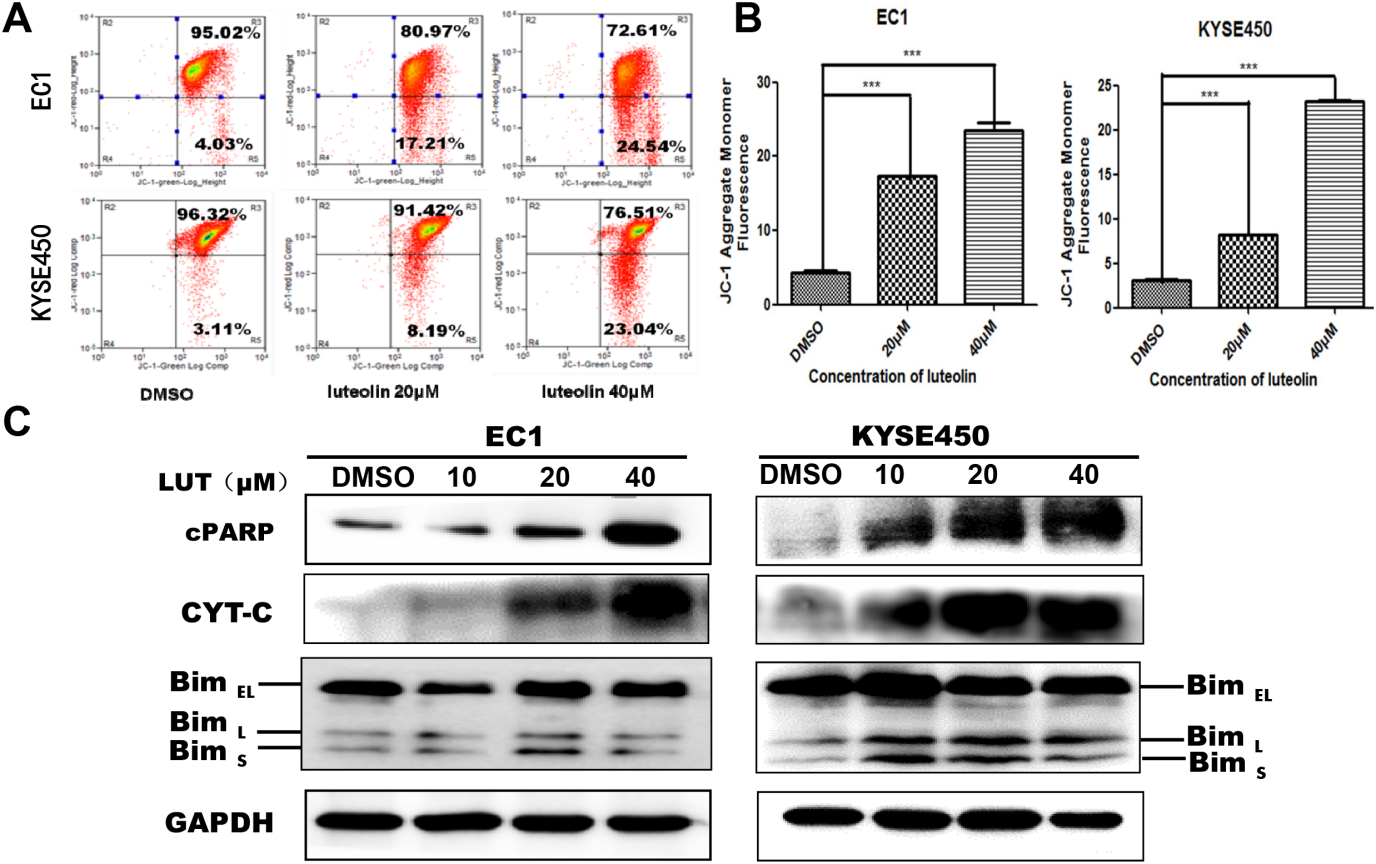
Mitochondrial membrane potential decreased and the expression mitochondrial apoptosis related proteins (cPARP, CYT-C, BimL and BimS) increased after being treated with luteolin **(A)** Mitochondria membrane potential decreased after EC1 and KYSE450 cells being treated with luteolin in a dose-dependent manner. **(B)** Dose-dependent changes in mean JC-1 fluorescence after cells being treated with luteolin for 48 h (mean ± SD from three independent experiments. ***p < 0.001). **(C)** Expression of mitochondrial apoptosis related proteins cPARP, CYT-C, Bim_EL_, Bim_L_ and Bim_S_ after EC1 and KYSE450 cells being treated with luteolin for 72h. GAPDH was used as reference.

JC-1 test results show that with luteolin concentration increased, mitochondrial membrane potential decreased (*p* < 0.05, Figure 4A, 4B). The results indicate that luteolin induced EC1 and KYSE450 cells apoptosis through mitochondrial pathway. Western blotting assay further revealed that the expression of Bim, CYT-C and cPARP were positively associated with the concentrations of luteolin used in current study (Figure 4C). Considering the evidence provided here, we propose that luteolin might induce apoptosis in EC1 and KYSE450 cells through mitochondria-dependent apoptotic pathway.

### Luteolin inhibited esophageal tumor growth in xenograft mouse models

**Figure 5:**
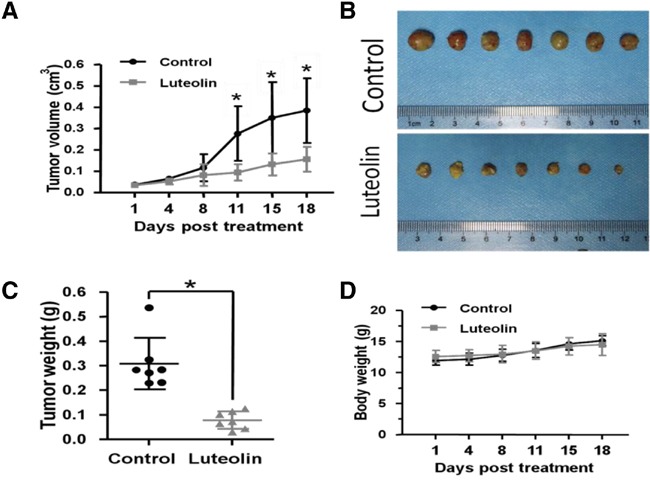
Luteolin inhibited tumor growth in ESCC xenograft mouse models **(A)** Tumor size change in control and luteolin treated groups. **(B)** Tumors harvested in control and luteolin treated groups. **(C)** Tumor weight in control and luteolin treated groups. **(D)** Body weight change in control and luteolin treated groups. (mean ± SD, *p < 0.05 (Student's t-test).

Finally, we validated the anti-tumor effects of luteolin in EC1 mouse xenograft models. After subcutaneous injection of EC1-E-GFP cells, fourteen nude mice were randomly divided into two even groups: control group (DMSO) and luteolin treated group. The results showed that growth of EC1-EGFP tumors was significantly inhibited by administration of luteolin (Figure 5A). Tumor size decreased, total tumor weight reduced by about 65% in luteolin treated group compared with control group (Figure 5B, 5C). No evidence of systemic toxicity was observed in luteolin treated mice (Figure 5D). Evidences provided here indicate that luteolin can prevent tumor formation and progression *in vivo*.

## DISCUSSION

Chemotherapy may be used to stall the development of ESCC and extend survival of ESCC patients [24, 25]. Cytotoxic agents, such as Cisplatin and Oxaliplatin, can be used as chemotherapeutic agents. However, cytotoxic chemotherapy is potentially hazardous because it may introduce side effects. In recent years, many researchers have paid attention to the utilization of natural extracts from plants as chemotherapeutic agents. Several studies have demonstrated that luteolin could induce cell cycle arrest and further inhibit proliferation and induce apoptosis in different types of cancer cells *in vitro*, including cell lines from hepatocellular carcinoma, cholangiocarcinoma, leukemia, thyroid cancer, breast cancer, prostate cancer, melanoma, lung cancer, oral squamous cancer, stomach cancer, gastric cancer, colon cancer, as well as ESCC and EAC [9, 17–19, 26–37].

In current study, we further confirmed the anti-proliferation effect of luteolin in EC1 and KYSE450 ESCC cells. Results from CCK-8 assay showed that luteolin could suppress the growth of four ESCC cell lines in a dose-dependent manner *in vitro* and compromise their colony-forming efficacies. This is consistent with findings from Zhang's and Wang's research groups [17–19]. In their studies, they reported that luteolin could induce growth inhibition in KYSE510 and Eca109 ESCC cell lines in a dose-dependent manner with estimated IC_50_ ranging from around 35 to 73 μM [18, 19]. In current study, we have similar findings with the IC_50_ of luteolin falling in the range of 20 to 60 μM. IC_50_ varies in different cancer cell lines. This indicates that different cancer cell lines might have different sensitivity to luteolin induced cell cycle arrest. Considering results from CCK-8 test and the degree of differentiation and origins of these four ESCC cell lines, EC1 and KYSE450 cells were chosen for further experiments. Results from flow cytometry revealed that EC1 and KYSE450 cells were arrested in G2/M phase after being treated with luteolin. Several studies have reported that luteolin can promote apoptosis but differentially induce cell cycle arrest in different types of cancer cell lines, either G1/S or G2/M arrest [9, 28–36, 38–40]. Given evidences provided here and by Zhang *et al* and Wang *et al*, it is postulated that luteolin can also differentially induce cell cycle arrest in different ESCC cell lines [18, 19].

In eukaryotes, two Cyclins (Cyclin A2 and Cyclin B1) and cyclin-dependent kinase 1 (CDK1) play central role in G2/M transition. P21^CIP1/ WAF1^ is a kind of CDK inhibitors (CKIs), which can regulate the activities of CDKs [41]. In cell lines from breast cancer, colon cancer, prostate cancer and lung cancer, several researchers have reported that flavonoid could induce cell cycle arrest in G2/M phase via p21^CIP1/ WAF1^ upregulation [18, 42–49]. In current study, we further confirmed this association. In addition, we also observed that p53 is overexpressed after luteolin treatment in both cell lines. Thus, we postulate that luteolin can induce cell cycle arrest in G2/M phase via upregulation of p21^CIP1/ WAF1^ and p53, and this can further induce apoptosis and inhibit cell proliferation in EC1 and KYSE450 cell lines.

There are two main pathways of apoptosis in mammals, intrinsic pathway, also called mitochondrial pathway, and extrinsic pathway. Mitochondrial pathway is the main target in developing anti-tumor drugs [50, 51]. P53, Bcl-2, Bax, caspase-3 and PARP are important proteins that participate in cell apoptosis mediated by mitochondrial pathway. And release of cytochrome C (CYT-C) is a key event of cell apoptosis. Results from flow cytometry indicated that luteolin could increase apoptotic rate in both cancer cell lines and the activities of caspase-3 also significantly increased in a dose-dependent manner. Caspase-3 plays a central role in the execution phase of cell apoptosis. Proteins from Bcl-2 family have an important role in the regulation of mitochondrial pathway and cytochrome C release. PARP can be inactivated by Caspase-3 cleavage and this will activate programmed cell death. In current study, results from Annexin V/PI double stain apoptosis assay and caspase-3 activities detection reveal that luteolin can induce apoptosis in EC1 and KYSE450 cells in a dose-dependent manner and this is associated with increased caspase-3 activities. Results from JC-1 kit assay revealed that membrane potential of mitochondrial decreased in a dose-dependent manner in these two cell lines after luteolin treatment. Results from Western Blot indicate that luteolin can upregulate Bim_L_ and Bim_S_ protein expression accompanied with CYT-C overexpression and cPARP cleavage. Based on these findings, we propose that luteolin can induce apoptosis in EC1 and KYSE450 cells through the mitochondrial pathway.

Furthermore, we constructed ESCC xenograft mouse models by subcutaneous injection of EC1-EGFP cells in nude mice. The results showed that tumor sizes in luteolin treated groups were significantly smaller than that in control groups. No significant cytotoxic side effects were observed since the body weight in both groups showed no difference. This is consistent with findings from other groups that luteolin can significantly inhibit cancer growth *in vivo* in xenograft mouse models for non-small cell lung cancer, squamous cell carcinoma of head and neck, breast cancer [37, 52–54].

In conclusion, current study has demonstrated that luteolin can induce cell cycle arrest in G2/M phase and apoptosis through mitochondrial pathway in EC1 and KYSE450 cell lines, and *in vivo* study has also demonstrated that luteolin can significantly inhibit the growth of tumor in ESCC xenograft mouse models. This has further elucidated the mechanisms of luteolin in ESCC treatment and prevention, and indicates that proper utilization of luteolin might be a practical approach in ESCC chemotherapy.

## MATERIALS AND METHODS

### Ethics statement

All experiments in present study were conducted in strict accordance with the recommendations in the Guide for the Care and Use of Laboratory Animals published by the US National Institutes of Health. The animal experiments were approved by the Animal Care and Use Committee at Zhengzhou University. The number of animals used was minimized, and all necessary precautions were taken to mitigate pain or suffering.

### *In vitro* experiment

#### Cell culture

The human Esophageal squamous carcinoma cell lines (EC1, EC9706, KYSE30 and KYSE450) were purchased from Shanghai cell bank (China Academy of Science) and cultured in DMEM medium (KYSE450/KYSE30), RPMI 1640 (EC1/EC9706) (Thermo Fisher Scientific, Beijing, China) which were supplemented with 10% fetal bovine serum (FBS, Gibco BRL Life technologies, Rockville, MD, USA), 100 U/ml penicillin and 100 μg/ml streptomycin (Sigma, St. Louis, MO) respectively under a humidified atmosphere of 5% CO_2_ at 37°C. DMSO was used as drug delivery vehicle in all the *in vitro* experiments, and the final concentration of DMSO in negative control was 0.1%. Cells were checked routinely and trypsinized until they reached 80-90% confluency. Luteolin was purchased from Enzo Life Sciences (Aladdin biological technology co. Ltd, Shanghai, China) and dissolved in DMSO solution prior to use.

### CCK-8 test

The cell viability was examined via cell counting kit-8 (CCK-8 kit, Keygen biotech Co., Ltd, Nanjing, China) according to the manufacturer's instruction [55]. Briefly, approximately 3 × 10^3^ cells were seeded in 100μl per well DMEM/RPMI 1640 in a 96-well plate. 100 μl luteolin at concentrations of 0, 10, 20, 40, 80 μM was added to the medium, and the cells were cultured for different time lengths (48 h and 72 h). Subsequently, 10 μl of the CCK-8 solution was added to each well and incubated at 37°C for another 2 h. The absorbance at 450 nm was measured on a spectrophotometric plate reader. Each group was repeated in three different wells.

### Plate colony formation assay

The ability of cells to form macroscopic colonies was determined by a plate colony formation assay [56]. Single cell suspensions derived from cells in the logarithmic phase were seeded in six-well plates (800/well), and luteolin at concentrations of 5, 10, 20, and 40 μM were used as treatment. After incubation at 37°C for 8 d, colonies were rinsed with PBS, fixed with 4% paraformaldehyde at room temperature for 30 min, and stained with 0.1% crystal violet (Sigma, St. Louis, MO, USA) for 30 min. Only clearly visible colonies (foci > 50 cells) were counted. The experiments were repeated three times.

### Flow cytometry for cell apoptosis and cell cycle distribution analysis

The influence of luteolin on apoptosis and cell cycle distribution were detected with Annexin V-FITC-/PI apoptosis kit (Keygen biotech Co., Ltd, Nanjing, China) and cell cycle kit (Keygen biotech Co., Ltd, Nanjing, China) separately according to the manufacturer's instructions. KYSE450 and EC1 cells were exposed to luteolin at concentrations of 0, 20, and 40 μM for 24 h or 72h. Then, cells were trypsinized, collected by centrifugation at 2000 rpm for 5 min and washed with PBS. Then corresponding regents and solution were added and incubated according to manufacturer's instructions respectively. At the end of incubation, cell apoptosis distribution was analyzed on a flow cytometer (Becton Dickinson, USA), and cell cycle data was analyzed with ModFit software (Verity Software House, Topsham, ME).

### Flow cytometry for cell caspase-3 activity and mitochondrial membrane potential (ΔΨm) analysis

Caspase-3 activity and mitochondrial membrane potential (ΔΨm) analysis were isolated using CaspGLOW™ Fluorescein Active Caspase-3 Staining Kit (BioVision co., USA) and mitochondrial membrane potential detection kit (JC-1) (Beyotime Biotechnology Inc., shanghai, China) according to the manufacturer's instructions respectively. Luteolin at concentrations of 20 and 40 μM were used as treatment for 72h. DMSO solution was used as control.

### Western blot

After being treated with luteolin at concentrations of 0, 10, 20, 40 μM for 72h, whole cell proteins were extracted with RIPA lysis buffer and protein concentrations were determined by the standard BCA method (Beyotime Institute of BiotechnologyInc., shanghai, China). Then whole cell proteins (50 μg) were separated by SDS-PAGE and transferred onto polyvinylidene difluoride membranes (Millipore Corp., Bedford, MA, USA). Membranes were blocked in TBS containing 0.1% Tween-20 and 5% dry milk, and probed with primary antibody overnight at 4°C. Primary antibody directly against Bim, cPARP, CYT-C, p21, p53 and GAPDH (all form Cell Signaling Technology, USA) were used at a dilution of 1:1000, followed by incubating with HRP-conjugated secondary antibodies for 2 h at room temperature. Signals were visualized using BeyoECL Plus (Millipore, Billerica, MA, USA) and captured using an Image Quant LAS-4000 (Fujifilm, Tokyo, Japan). All the experiments were repeated three times. The protein quantification of the Western blot results was normalized to GAPDH and then compared to the control group.

### Xenograft experiments

Fourteen six-week-old female BALB/C-nu mice with green fluorescent protein marker of human esophageal cancer (EC1-E-GFP) were used in this study to test the therapeutic effects of luteolin *in vivo*. They were cultured in pathogen-free sterile conditions with continuous access to sterile food and water. For tumorigenicity assays, 5×10^6^/100 μl EC1-EGFP cells were subcutaneously injected into the upper portion of the right hind limb of BALB/c Nude mice to construct ESCC xenograft models. After tumor grows, the xenograft mouse models were randomly divided into two even groups, control and treatment (50mg/kg luteolin) groups. Mice in the control group were injected with HPBCD and mice in the treatment group were injected with luteolin (50mg/kg) to enterocoelia as intervention. Tumors were observed by FluorVivo (INDEC BioSystems, CA, USA) and size was measured using calipers every 3 days. Cervical dislocation was conducted after mice being euthanized by Isoflurane (Abbot Laboratories Ltd., North Chicago, IL) inhalation 18 days after luteolin intervention. At the end of this experiment, tumors were harvested and weighed. The condition of mice was observed every day and the mice were weighed twice a week.

### Statistical analysis

All statistical analyses were conducted by using the SPSS 19.0 statistical software (IBM, Armonk, New York). Results are expressed as means ± SD. Two-tailed independent-samples Student's t-test was used to determine the difference between two mean values, and two-sided p values less than 0.05 were considered statistically significant.
